# Protein Similarity Networks Reveal Relationships among Sequence, Structure, and Function within the Cupin Superfamily

**DOI:** 10.1371/journal.pone.0074477

**Published:** 2013-09-06

**Authors:** Richard Uberto, Ellen W. Moomaw

**Affiliations:** Department of Chemistry and Biochemistry, Kennesaw State University, Kennesaw, Georgia, United States of America; Oak Ridge National Laboratory, United States of America

## Abstract

The cupin superfamily is extremely diverse and includes catalytically inactive seed storage proteins, sugar-binding metal-independent epimerases, and metal-dependent enzymes possessing dioxygenase, decarboxylase, and other activities. Although numerous proteins of this superfamily have been structurally characterized, the functions of many of them have not been experimentally determined. We report the first use of protein similarity networks (PSNs) to visualize trends of sequence and structure in order to make functional inferences in this remarkably diverse superfamily. PSNs provide a way to visualize relatedness of structure and sequence among a given set of proteins. Structure- and sequence-based clustering of cupin members reflects functional clustering. Networks based only on cupin domains and networks based on the whole proteins provide complementary information. Domain-clustering supports phylogenetic conclusions that the N- and C-terminal domains of bicupin proteins evolved independently. Interestingly, although many functionally similar enzymatic cupin members bind the same active site metal ion, the structure and sequence clustering does not correlate with the identity of the bound metal. It is anticipated that the application of PSNs to this superfamily will inform experimental work and influence the functional annotation of databases.

## Introduction

The cupin superfamily of proteins possesses remarkable functional diversity with representatives found in Archaea, Eubacteria, and Eukaryota [1], [2], [3], [4], [5]. The identification of the cupin superfamily was originally based on the recognition that the wheat protein germin shared a nine amino acid sequence with another protein, spherulin, produced by the slime mold *Physarum polycephalun* during starvation [3]. This sequence similarity was also observed in a number of seed storage proteins called germin-like proteins (GLPs). Knowledge of the three dimensional structures of these proteins led to the collective name “cupin” on the basis of their β-barrel shape (“cupa” means “small barrel” in Latin) [4]. Characteristic features of proteins with this fold include high thermal stability and resistance to proteases. These features are consistent with their high degree of subunit contacts, hydrophobic interactions, and short loops. The cupin domain was originally described as two conserved motifs, each composed of two β-strands [2], [5]. Motif 1 was designated as G(X)_5_HXH(X)_3,4_E(X)_6_G and Motif 2 as G(X)_5_PXG(X)_2_H(X)_3_N. The two motifs are separated by an intermotif region which ranges from 15 to 50 amino acids long. With more sequences analyzed, it became clear that the primary sequence of the two motifs is not as highly conserved as previously thought [1], [6].

**Figure 1:**
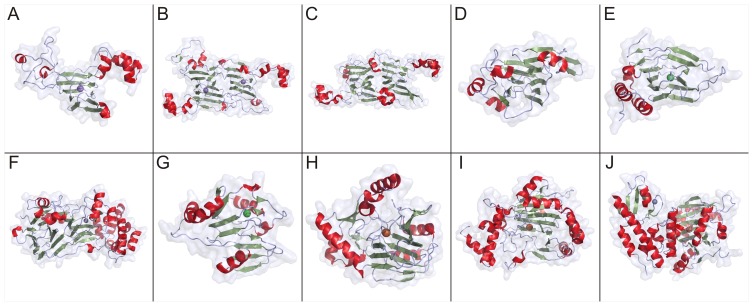
Structures of representative of members of the cupin superfamily. A. oxalate oxidase (PDB code: 2et1) [72], B. oxalate decarboxylase (PDB code: 1uw8) [43], C. seed storage protein Ara h (PDB code: 3s7i) [39], D. NovW, a 4-keto-6-deoxy sugar epimerase (PDB code: 2c0z) [71], E. cysteine dioxygenase (PDB code: 2q4s) [48], F. phosphomannose isomerase (PDB code: 1 pmi) [53] G. acireductone dioxygenase (PDB code: 1 zrr) [62] H. taurine/alpha-ketoglutarate dioxygenase (PDB code: 1os7) [73], I. hypoxia-inducible factor 1-alpha inhibitor (PBD code: 2y0i) [74], J. lysine-specific demethylase 6B (PDB code: 2 xue) [75]. β-sheets are shown in green, α-helices are shown in red, and random coils are shown in grey. Spheres represent bound metal ions. Figures were generated using Pymol (The PyMOL Molecular Graphics System, Schrödinger, LLC).

**Figure 2:**
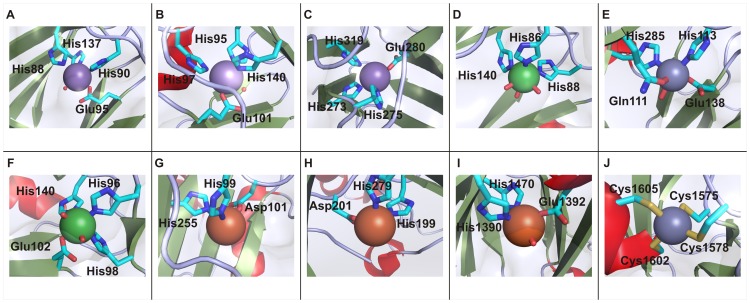
Metal-binding sites of representative members of the cupin superfamily. A. Mn ion of oxalate oxidase coordinated by His88, His90, Glu95, and His137 (PDB code: 2et1) [72], B. N-terminal Mn ion of oxalate decarboxylase coordinated by His95, His97, Glu101, and His140 (PDB code: 1uw8) [43], C. C-terminal Mn ion of oxalate decarboxylase coordinated by His273, His275, Glu280, and His319 (PDB code: 1uw8) [43], D. Ni ion of cysteine dioxygenase coordinated by His86, His88, and His140 (PDB code: 2q4s) [48], E. Zn ion of phosphomannose isommerase coordinated by Gln111, His113, Glu138, and His285 (PDB code: 1 pmi) [53] F. Ni ion of acireductone dioxygenase coordinated by His96, His98, Glu102, and His140 (PDB code: 1 zrr) [62], G. Fe ion of taurine/alphaketoglutarate dioxygenase coordinated by His99, Asp101, and His255 (PDB code: 1os7) [73], H. Fe ion of hypoxia-inducible factor 1-alpha inhibitor coordinated by His199, Asp201, and His279 (PBD code: 2y0i) [74], I. Fe ion of lysine-specific demethylase 6B coordinated by His1390, Glu1392, and His1470 (PDB code: 2 xue) [75], J. Zn ion of lysine-specific demethylase 6B coordinated by Cys1575, Cys1578, Cys1602, and Cys1605 (not part of the cupin domain) (PDB code: 2 xue) [75]. β-sheets are shown in green, α-helices are shown in red, and random coils are shown in grey. Spheres represent bound metal ions. Figures were generated using Pymol (The PyMOL Molecular Graphics System, Schrödinger, LLC).

Genomic enzymology; large-scale sequence, functional, and structural databases; and inferences from molecular evolution have informed family, superfamily, and suprafamily designations [7], [8], [9], [10]. Enzyme families are relatively recently-diverged groups of enzymes that share similar three-dimensional structures and functions. Enzyme superfamilies consist of enzymes that diverged earlier and possess fewer common elements but share a common mechanistic attribute, while suprafamilies share conserved residues but do not share a common mechanistic attribute [8], [11]. Inconsistencies exist in the usage of the term ‘cupin’. According to the Structural Classification of Proteins (SCOP) database [12], [13], [14], cupin proteins are members of the ‘RmlC-like Cupins’ superfamily within the double-stranded β-helix (DSBH) multicatalytic fold [15]. The term ‘cupin superfamily’ has often been used to refer to those proteins defined by the SCOP database as well as the 2-oxoglutarate-Fe^2+^-dependent dioxygenase superfamily that also possesses the DSBH fold [1], [5]. However defined, the cupin superfamily is extremely diverse and includes catalytically inactive seed storage and sugar-binding metal-independent proteins as well as metal-dependent enzymes possessing dioxygenase, decarboxylase, and other activities [10]. Figure 1 (A–J) shows the structures of 10 representative proteins designated as cupins in the Pfam database. Although the majority of enzymatic cupins contain iron as an active site metal, other members may contain nickel, zinc, manganese, cobalt, or copper. Each cofactor allows a different type of chemistry to occur within the conserved tertiary structure. Proposed reaction mechanisms of the metal-dependent cupins generally involve sequential binding of the substrate and dioxygen to the catalytic metal cation [16]. Figure 2 (A-J) shows the metal-binding sites of 8 representative members of the enzymatic cupins.

The DSBH (also referred to as jelly roll) topology is most often composed of eight β-strands that form a β-sandwich structure comprised of two four-stranded antiparallel β-sheets. Although four forms are possible (two hands of the helix and two directions to trace the structure), only one form (right-handed class I) is prevalent in nature [17]. Ancestral cupins can be evolutionarily reconstructed as simple, small molecule-binding domains that likely bound sugars and cyclic nucleotides [5], [7], [18]. These sugar-binding domains later gave rise to sugar-modifying domains such as isomerases and epimerases [19]. Analyses of the evolution of the fold suggest that a set of conserved histidine residues employed in sugar-binding in the ancestral non-enzymatic domain evolved into the metal-coordinating histidine residues observed in oxalate oxidase (Figures 1A and 2A) [19] and oxalate decarboxylase (Figures 1B, 2B, and 2C) [20]. Another lineage of DSBH domains acquired a new set of conserved residues with the ability to bind 2-oxoglutarate which gave rise to the 2-oxoglutarate-Fe^2+^-dependent dioxygenases [7], [21].

The exponential growth of structural information for proteins provides abundant material for the analysis of how protein structure informs biological function and chemical reactivity. Babbitt *et al.* recognized the need to associate structure and sequence information with biological function in ways that are accessible to both experimental and computational biologists. They presented protein similarity networks (PSNs) to fulfill this need [22]. PSNs have contributed to our understanding of a number of large groups of proteins including the enolase superfamily [23], the ePK-like superfamily [24], glutathione transferases [25], [26], strictosidine synthase-like proteins [27], cysteine peptidases [28], and proteins used in algal metal transport [29]. These studies have yielded meaningful insights, validated PSN methodology, and provided an understanding of the caveats and limitations of PSNs. PSNs are complementary to phylogenetic studies and provide different and new information compared to other methods relating structures and sequences. It has been noted that protein similarity networks are most compelling when painted with functional or structural information that is orthogonal to the data used to generate the networks [22].

The Pfam database [30] lists 112,082 cupin sequences represented in 6529 species and 945 associated protein structures. This represents a greater than 10-fold increase in the number of sequences in only four years [15]. Protein similarity networks are a way to visualize large-scale computational analyses of sequence and structure among a given set of proteins [22], [24] and have been used to guide experimental design and data interpretation [27], [31]. In this work we describe the first visualization of sequence and structure data of the cupin superfamily using PSNs. The PSNs were built using open source software programs Pythoscape [32] and Cytoscape [33]. In these networks the protein structures (or sequences) are represented as nodes, and their similarities to each other are represented as edges. A value of this work lies in displaying nodes by particular attributes. Node attributes that can be gathered automatically through Pythoscape include sequence, organism, description, and identification codes. Other manually-retrieved attributes may be inputted, such as the presence of a catalytic motif or reaction intermediates. Overlaying attribute information onto clusters allows visualization of the relationship between structure and function. There exists a large amount of structure and sequence information for cupin superfamily members for which there has been no experimental work conducted. By facilitating the prediction of the functions of these proteins, PSNs may be used to guide experimental inquiry into the cupin superfamily.

## Results and Discussion

The Pfam database [30] contains 484 unique cupin structures. For visual simplicity, only a single representative structure of a protein with multiple structures was used in the networks. This reduced the networks to 183 structures, which are listed in Supporting Information (Tables S1–S7 in File S1). Of the 183 PDB structures representing unique members of the cupin superfamily, 76 bind no metal, 49 bind iron, 18 bind nickel, 16 bind zinc, 10 bind manganese, 1 binds cadmium (protein yhhW, PDB code: 1tq5) [34], 1 binds copper (quercetin dioxygenase from *Aspergillus japonicus*, PDB code:1 juh) [35], 1 binds mercury (protein YhcH, PDB code: 1 jop) [36], and 11 bind multiple metals.

**Figure 3:**
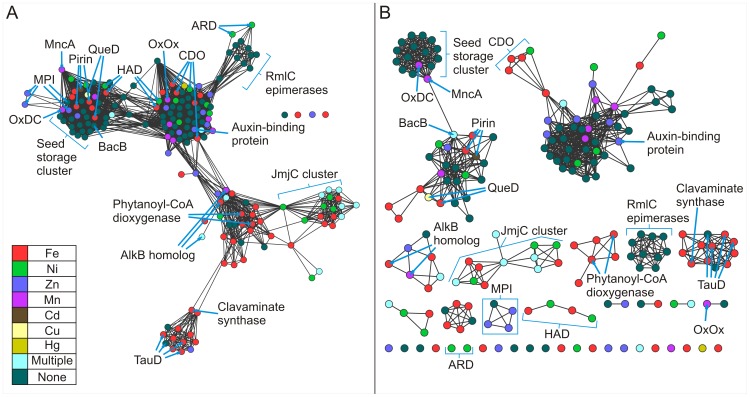
Structure similarity networks of the cupin protein stuctures colored by metal ligand. Pairwise similarities for a non-redundant set of 183 structures from the Pfam cupin clan (CL0029) were calculated using TM-align [37]. Each node represents a structure. Nodes were arranged using the yfiles organic layout of of Cytoscape version 3.0. A. Edges between nodes were drawn only if the average TM-score >0.53 for that edge. At this cutoff, the average r.m.s.d. is 2.91 Å with an average of 158.0 Cα atoms aligned. B. Edges between nodes were drawn only if the average TM-score >0.65 for that edge. At this cutoff, the average r.m.s.d. is 2.44 Å with an average of 185.4 Cα atoms aligned.

Figure 3 shows pairwise similarities for this non-redundant set of structures calculated using TM-align [37] (See Methods) at two different stringency thresholds. This permits alternative views of the same structural relationships. These networks are painted according to the identity of the bound metal in the structure. In the less stringent network (Figure 3A), all but four structures have connections to a single large constellation of structures. This constellation partitions off into smaller clusters of structures in the more stringent network (Figure 3B). It can be observed that often proteins of the same function cluster together. For example, at the higher stringency the catalytically inactive seed storage proteins cluster together with two metal-binding cupins, oxalate decarboxylase and the MncA protein. Other clusters analyzed in this work include the cysteine dioxygenases (CDOs), the Jumonji C domain-containing proteins (JmjC), and the RmlC epimerases.

### Seed Storage Proteins Cluster Together

**Figure 4:**
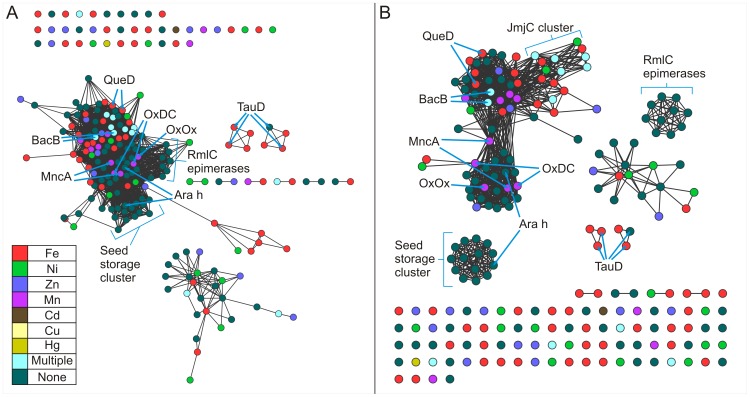
Structure similarity networks of cupin domains colored by metal ligand. Pairwise similarities for a non-redundant set of 213 domains from the Pfam cupin clan (CL0029) were calculated using TM-align [37]. Each node represents a domain. Nodes were arranged using the yfiles organic layout of of Cytoscape version 3.0. A. Edges between nodes were drawn only if the average TM-score >0.53 for that edge. At this cutoff, the average r.m.s.d. is 1.73 Å with 74.2 Cα atoms aligned. B. Edges between nodes were drawn only if the average TM-score >0.65 for that edge. At this cutoff, the average r.m.s.d. is 1.42 Å with 80.1 Cαatoms aligned.

The seed storage proteins cluster together at the lower threshold (Figure 3A), but share edges with other protein structures. At the higher threshold (Figure 3B), however, these proteins partition exclusively. Members of this cluster contain two cupin domains and are, therefore, classified as bicupins. The majority of the members of this group contain no metal and include procruciferin (PDB code: 3 kgl) [38], Ara h (PDB code: 3s7i) [39], phaseolin (PDB code: 2 phl) [40], beta-conglycinin (PDB code: 1 uij) [41], and proglycinin (PDB code: 1 fxz) [42]. The two metal-containing members of this cluster are oxalate decarboxylase (PDB code: 1uw8) [43] and MncA (PDB code: 2 vqa) [44] with both incorporating manganese. The architecture of this group is represented in Figure 1B by oxalate decarboxylase (OxDC) and in Figure 1C by Ara h, of interest as a major peanut allergen. The two nearly identical Mn-binding sites of OxDC are shown in Figures 2A and 2B. Previous work with protein similarity networks in general had indicated that the clustering of proteins does not change dramatically whether the domain in common is isolated or is a component of a larger multi-domain complex [22], and this is our observation also. However, we were concerned that monocupins were generally segregated from bicupins in the network built using entire structures. In an effort to explore this possible limitation, we constructed networks of isolated cupin domains for the structures used in Figure 3. Figures 4A and 4B show the cupin domain networks at the same stringencies as those in Figures 3A and 3B, respectively, also colored by metal ligand. At both stringencies, monocupin oxalate oxidase (OxOx) (Figures 1A and 2A) clusters with both the N-terminal and C-terminal cupin domains of bicupin OxDC. At the higher stringency (Figure 4B), OxDC (both domains), MncA (both domains), and OxOx partition away from the group labeled “seed storage cluster.” Interestingly, many of the seed storage proteins such as Ara h split one domain to the “seed storage cluster” and one domain remains in the group containing OxDC, MncA, and OxOx. This observation is consistent with the conclusions from phylogenetic analyses that the N-and C-terminal domains of many bicupins arose through independent evolutionary events [15] whereas others such as OxDC arose through gene duplication events [2].

**Figure 5:**
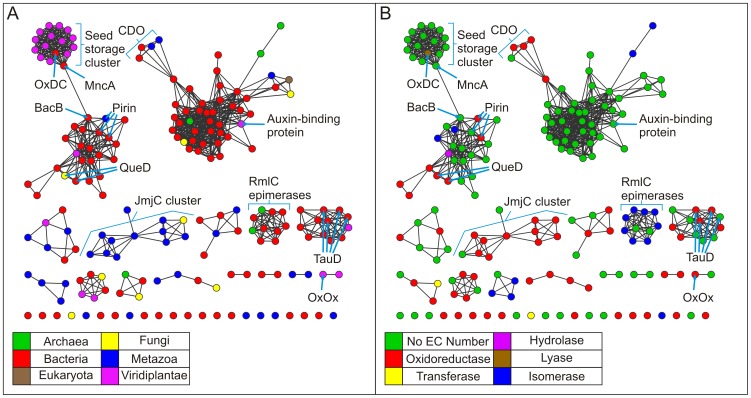
Structure similarity networks colored by species and function. This is the same network as in 3B but colored according to A. species and B. function.

**Figure 6:**
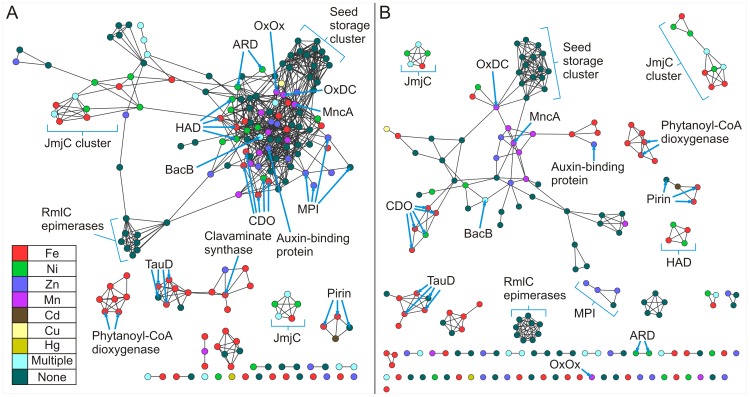
Sequence similarity networks colored by metal ligand. Networks were generated by all-by all BLAST comparisons of the 183 sequences corresponding to the unique cupin structures shown in Figure 3. Nodes were arranged using the yfiles organic layout of of Cytoscape version 3.0. A. Edges between nodes are drawn only if the E-value is better than of 1E-3.5. At this cutoff, edges at this threshold represent alignments with a median 32.1% identity over 93 residues. B. Edges between nodes are drawn only if the E-value is better than of 1E-6.0. At this cutoff, edges at this threshold represent alignments with a median 36.2% identity over 185 residues.

Figure 5 reproduces the same network as Figure 3B but is painted by kingdom (Figure 5A) and function (Figure 5B). Figure 5A illustrates that OxDC and MncA are bacterial proteins and the only non-plant members of the seed storage cluster. OxDC is a Mn-dependent enzyme that catalyzes the carbon-carbon bond cleavage of oxalate to yield carbon dioxide and formate in a reaction with no net change in oxidation state (the only lyase represented in Figure 5B) [45]. MncA is the most abundant Mn-containing protein in cyanobateria *Synechocystis* PCC 6803 and played a key role in a study that elucidated a mechanism whereby the compartment in which a protein is folded overrides its binding preference to control its metal content [44]. MncA is first neighbors with (shares edges) in the structure networks (Figures 3A, 3B, 5A, and 5B) with all members of the seed storage cluster as well as BacB (PDB code: 3h7j) [46] in an adjacent cluster. BacB binds both Fe and Co, has been shown to play a role in the synthesis of bacilysin, and clusters with pirins and quercetin dioxygenases (see below). Networks based on sequence (Figures 6A and 6B) similarly cluster the seed storage proteins which share edges with OxDC and MncA.

### Enzymes Cluster Based on Function

The structure network at a more permissive threshold (Figure 3A) clearly shows clustering correlated with function. For example, the four cysteine dioxygenases, which catalyzes the oxidation of the L-cysteine to its sulfinic acid by incorporating both of the oxygen atoms from O_2_ into the product, are part of a larger cluster also containing oxalate oxidase (OxOx) and auxin-binding protein at the lower stringency (Figure 3A). In the more stringent network (Figure 3B), the CDO structures (*Homo sapiens*, PDB code: 2ic1 [47]; *Ralstonia eutropha,* PDB code: 2gm6; *Mus musculus* PDB code: 2q4s [48]; and *Pseudomonas aeruginosa* PDB code: 3 uss) share first neighbor status with each other only and are connected to the larger cluster through the putative CDO from *Bacillus subtilis* (PDB code: 3 eqe). These monocupins are represented in Figure 1E by the *Mus musculus* (mouse) enzyme. The metal ion in this structure is Ni(II) and is coordinated by three conserved residues (His 86, His88, and His140) as shown in Figure 2D. Biochemical characterization of this protein has shown that iron is required for catalytic activity [16]. CDOs cluster by function when the entire structures are compared (Figure 3), but remain separate due to the smaller region of alignment when compared at the domain level at the same stringency (Figure 4, data not indicated with arrows). The majority of cupins for which there is structural information are from bacterial organisms. The CDOs cluster by function in the sequence networks (Figure 6). These networks confirm and extend previous observations that members of the cupin superfamily cluster by function when the function is defined at a fine level, such as the oxidation of cysteine but do not do so when function is defined more broadly [15]. For example, the Fe-containing 2-oxoglutarate-dependent dioxygenases such as phytanoyl-CoA dioxygenase, taurine dioxygenase (TauD) (Figures 1H and 2G), and lysine-specific demethylases (JmjC cluster, see below) are separated in both the structure and sequence networks. Furthermore, the networks painted by metal ion (Figures 3, 4, and 6) show that neither structure-based nor sequence-based clustering correlates with the identity of the active site metal.

Structures are available for 3-hydroxyanthranilate-3,4-dioxygenase (HAD) from four species. HAD from *Cupriavidus metallidurans* (formerly *Ralstonia metallidurans*) (PDB code:1 yfu) [49], [50] and *Bos taurus* (PDB code: 3fe5) [51] contains iron, while HAD from *Saccharomyces cerevisiae* (PDB code:1 zvf) and *Homo sapiens* (PDB code: 2 qnk) contains nickel. These four structures group together by function in the network generated using the whole protein (Figure 3) but not when the cupin domain alone is used to generate the network (Figure 4). The HADs cluster by function in the sequence networks (Figure 6). Similarly, four structures are available for mannose-6-phosphate isomerases (MPIs) (Figures 1F and 2E). These structures segregate exclusively at the more stringent thresholds of the whole protein structure network (Figure 3B) and sequence network (Figure 6B), but not in the domain networks (Figure 4). Of these, three contain Zn (*Bacillus subtilis*, PDB code: 1 qwr; *Salmonella typhimurium*, PDB code: 3h1m [52]; and *Candida albicans*, PDB code: 1 pmi [53]).One putative PMI was crystallized with no metal present (*Archaeoglobus fulgidus*, PDB code:1zx5).

Quercetin 2,3-dioxygenase (QueD) catalyzes the insertion of molecular oxygen into polyphenolic flavonols. Structural information is available for quercetin 2,3-dioxygenases from *Aspergillus japonicus* (PDB code: 1 juh, [35]) and *Bacillus subtilis*, (PDB code: 1y3t [54]). QueD is able to employ a variety of metals in catalysis [54], [55], [56]. Incubation of apoprotein with transition metal ions has been done to examine the effects of different metal ions on enzymatic activity. Results yielded an activity profile with trends that were consistent with the Irving-Williams metal ion series based on the stability of metal ion complexes [57]. Data suggest that Mn(II) is the preferred cofactor for the enzyme [55]. Pirins have been shown to possess QueD activity [34], and structural information is available for pirins from *Escherichia coli* (PDB code: 1tq5) [34], *Homo sapiens* (PDB code: 1j1l), and *Geobacillus kaustophilus* (PDB code: 2p17). The QueDs and pirins are members of the same larger cluster in the structure-based networks (Figure 3). Consistent with previous analyses and observations [15], the pirin N- and C- terminal domains do not share edges with each other in the domain-based networks, suggesting an independent evolution of these domains in bicupins. This is in contrast to the N- and C- terminal domain clustering of bicupins such as OxDC, QueD, and BacB from *Bacillis subilis* where gene duplication events are proposed to play a large role in i) increasing the size of the genome and ii) producing bicupin architectures [2].

Acireductone dioxygenase (ARD) is a particularly interesting example of how the cupin scaffold can be used to catalyze different reactions in that this enzyme catalyzes different reactions depending on the type of metal ion bound in the active site [58], [59]. ARD serves as a branch point in the methionine salvage pathway. This pathway returns the γ-thiomethyl group of methylthioadenosine to methionine [60]. The overexpression of the ARD gene in *E. coli* yields two enzymes with different activities that are able to be separated by conventional chromatographic techniques of ion exchange and hydrophobic interaction resins. One enzyme catalyzes the on-pathway oxidation of acireductone to ketoacid and formate; the other enzyme catalyzes an off-pathway oxidation with formation of CO. The only difference between the two enzymes (Fe-ARD and Ni-ARD) was the metal bound in the active sites [58]. There is structural information available for ARD from *Mus musculus* (PDB code: 1vr3) [61] and *Klebsiella pneumonia* (PDB code: 1 zrr) (Figures 1G and 2F) [62]. In the more permissive structure network (Figure 3A) both ARD structures, like the RmlC epimerases, are connected to the larger cluster through dTDP-6-deoxy-3,4-keto-hexulose (PDB code: 2pa7) [63] but lose all edges in the more stringent network (Figure 3B). Similarly, in the more permissive sequence network (Figure 6A) the ARD structures share an edge with each other as well as other proteins but partition off into an independent doublet (sharing a single edge) under a more stringent threshold (Figure 6B).

That members of the cupin superfamily cluster by function when the function is defined at a fine level allows one to make functional inferences which may provide a starting point for biochemical investigations. For example, nearest neighbor analysis of the protein product of AT3G21360 of *Arabidopsis thaliana* (PBD code: 1y0z) [64] in the more stringent structure network (Figure 3B, not shown with arrows) suggests that the protein product of AT3G21360 may be an alpha ketoglutarate-dependent TauD. This inference is further supported by the exclusive partitioning of this protein with PDB codes “3v15” and “10s7” (both confirmed TauDs) in the more stringent domain-based network (Figure 4B). Similarly, the uncharacterized *Escherichia coli* protein yeaR (PDB code: 3bb6) shares an edge exclusively with the *Vibrio fisheri* tellurite resistance protein B (PDB code: 3dl3) in both the more stringent structure network (Figure 3B) and the less stringent domain-based network (Figure 4A) (not shown with arrows in either network).

### JmjC Proteins

The Jumonji C domain-containing proteins are a subfamily of the Fe(II)/2-oxoglutarate-dependent oxygenases. Sequence similarity between JmjC and cupin metalloenzyme domains allowed the prediction of active-site residues in JmjC domains and provided the insights that guided the experimental determination of the reactions catalyzed by these enzymes [65]. The JmjC domain-containing factor inhibiting hypoxia (FIH) (Figures 1I and 2H) was identified as an asparaginyl hydroxylase that transcriptionally regulates the activity of hypoxia-inducible factor (HIF) [66]. JmjC domain-containing proteins were proposed to function as histone demethylases in regulating chromatin structure [67] and many of the JmjC domain-containing enzymes have been shown to comprise the largest class of histone demethylases that catalyze lysine demethylation of mono-, di-, and trimethylated lysine residues through the formation of a hemiaminal intermediate that yields the demethylated product and formaldehyde [68], [69]. The lysine demethylases are represented by lysine-specific demethylase 6B in Figures 1J, 2I, and 2J. Additionally, JmjC domain-containing enzymes have been identified that have RNA hydroxylase activity [21], [70].

At the time of this writing, the cupin clan of the Pfam database contains structures of 13 unique JmjC domain-containing proteins (Table 1). Most (12) of these are given the Pfam family designation ‘JmjC’. These 12 structures form an exclusive cluster together in Figure 3B. The other JmjC domain-containing protein structure (PDB code: 3ld8) is designated ‘cupin 8’ in Pfam along with an additional three proteins that have structural information available (Table 1). These four structures form an exclusive cluster in Figure 3B (not labeled under the AlkB-containing cluster). Finally, the three proteins with structures available designated ‘cupin 4’ in Pfam have been included in Table 1 for comparison. In Figure 3B these structures exist as a pair of nodes and as a single node. In the more stringent sequence similarity network (Figure 6B), six JmjC domain-containing protein structures and the ‘cupin 8’ structures cluster together (PDB codes: 2yu2, 3k3n, 3n9l, 3pu8, 3u78, 3ld8, 4 aap, 3a16, 2y0i), while five JmjC domain-containing protein structures form an exclusive cluster (PDB codes: 2gp3, 2w2i, 2 xml, 3 dxu, 3 opt). The three ‘cupin 4’ structures partition into an exclusive triad. These networks capture a current snapshot of relationships within this subfamily and can be used to update relationships and guide experimental design as new structures become available.

**Table 1:** JmjC protein clusters.

Protein	Species	PDB code	Pfam family	Metal ion(s)	Ref.
Lysine-specific demethylase 6B	*Homo sapiens*	2 xue	JmjC	Fe, Zn	[75]
JmjC domain-containing histone demethylation protein 1^a^	*Homo sapiens*	2yu2	JmjC	Fe	
Lysine-specific demethylase 6A	*Homo sapiens*	3 avr	JmjC	Ni, Zn	[76]
catalytic domain of PHD finger protein 8	*Homo sapiens*	3k3n	JmjC	Fe	[77]
Lysine-specific demethylase 7	*Caenorhabditis elegans*	3n9l	JmjC	Fe, Zn	[78]
PHD finger protein 2	*Homo sapiens*	3pu8	JmjC	Fe	[79]
Lysine-specific demethylase 7	*Homo sapiens*	3u78	JmjC	Ni	[80]
Jumonji domain-containing protein 2A	*Homo sapiens*	2gp3	JmjC	Fe, Ni	[81]
Lysine-specific demethylase 4E	*Homo sapiens*	2w2i	JmjC	Ni	
JMJD2C catalytic domain	*Homo sapiens*	2 xml	JmjC	Ni, Zn	
JmjC domain-containing histone demethylation protein 3D	*Homo sapiens*	3 dxu	JmjC	Fe	
DNA damage-responsive transcriptional repressor RPH1	*Saccharomyces cerevisiae*	3 opt	JmjC	Ni	[82]
Bifunctional arginine demethylase and lysyl-hydroxylase JMJD6	*Homo sapiens*	3ld8	cupin 8	Fe, Hg	[83]
Lysine-specific demethylase 8	*Homo sapiens*	4 aap	cupin 8	Ni	
JmjC domain-containing protein C2orf60	*Homo sapiens*	3al6	cupin 8	Ni	[84]
factor inhibiting HIF1	*Caenorhabditis elegans*	2y0i	cupin 8	Fe	[74]
Putative asparaginyl hydroxylase	*Homo sapiens*	1 vrb	cupin 4	Fe	
Lysine-specific demethylase NO66	*Homo sapiens*	4 diq	cupin 4	Ni	
MYC-induced nuclear antigen	*Homo sapiens*	2 xdv	cupin 4	Cd, Ni, Mn	

### RmlC Epimerases

The RmlC epimerases in Figure 3A cluster together but share edges with other protein structures. At a more stringent threshold (Figure 3B), however, these same 10 structures (Table 2) cluster independently from other structures. The epimerases are monocupins. Members of this group do not bind a metal ion and are represented in Figure 1D by NovW, a 4-keto-6-deoxy sugar epimerase (PDB code: 2c0z) [71]. This grouping is in agreement with a published structure-based phylogenetic analysis of the cupin superfamily generated using a structure dissimilarity matrix through pairwise structure-based alignment of 52 cupin proteins [15]. Our analysis includes 2 additional proteins whose structures were solved after the phylogenetic analysis was published. The network in Figure 3B clearly groups the functionally similar RmlC epimerases together as does the phylogenetic analysis, providing further validation that PSNs recapitulate much of the information present in phylogenetic trees [22]. When the network is constructed of isolated domains, the 10 monocupin RmlC epimerase domains form the same cluster as in the whole protein network (Figure 4B). Furthermore, a sequence-based network clusters 9 of the 10 epimerases together only when edges between nodes are drawn if the E-value is better than of 1E-6.0 (Figure 6B). In this case the enzyme from *Aneurinibacillus thermoaerophilus* is excluded from the cluster.

**Table 2:** The RmlC epimerase cluster.

Protein	Species	PDB code	# of chains	Metal ion	Ref.
dTDP-4-keto-6-deoxy-d-hexulose 3, 5-epimerase	*Methanobaterium thermoautotrophicum*	1ep0	1	none	[85]
4-keto-6-deoxy sugar epimerase (NovW)	*Streptomyces spheroides*	2c0z	1	none	[86], [87]
dTDP-6-deoxy-D-xylo-4-hexulose 3,5-epimerase	*Pseudomonas aeruginosa*	1 rtv	1	none	[88]
dTDP-4-dehydrorhamnose 3,5 epimerase	*Mycobacterium tuberculosis*	1 upi	1	none	[88], [89]
dTDP-4-keto-6-deoxy-glucose-5 epimerase EvaD	*Amycolatopsis orientalis*	1 ofn	2	none	[90]
dTDP-4-dehydrorhamnose 3,5 epimerase	*Sulfolobus tokodaii*	1 wlt	2	none	
dTDP-4-dehydrorhamnose 3,5 epimerase	*Streptococcus suis*	1 nxm	2	none	[91]
dTDP-4-dehydrorhamnose 3,5 epimerase	*Salmonella typhimurium*	1 dzr	2	none	[92]
dTDP-4-dehydrorhamnose 3,5 epimerase	*Bacillus anthracis*	3 ryk	2	none	
dTDP-6-deoxy-3,4-keto-hexulose isomerase	*Aneurinibacillus thermoaerophilus*	2pa7	2	none	[63]

### Conclusions

Protein similarity networks (structure and sequence) of the cupin superfamily recapitulate and complement phylogenetic studies. Structure- and sequence-based clustering of cupin members reflects functional clustering. Networks based only on cupin domains and networks based on the whole proteins provide complementary information. Domain-clustering supports phylogenetic conclusions that the N- and C- terminal domains of bicupin proteins evolved independently. Interestingly, although many functionally similar enzymatic cupin members bind the same active site metal ion, the structure and sequence clustering does not correlate with the identity of the bound metal. PSNs are expected to be a valuable tool for directing experimental work and for predicting the functions of uncharacterized members of the cupin superfamily.

## Methods

### Dataset Curation

All structures associated with the PFAM cupin clan (CL0029) were downloaded 31 March 2013. There were 945 chains from 484 different structures (many structures had multiple chains). However, many structures were that of the same protein, a problem that could cause an undue distortion of network topology. Duplicates were manually removed to avoid this issue, reducing the set of proteins to 183 unique structures. The structure used to represent duplicate structures was selected arbitrarily. Initially the structures of individual chains of whole protein structures were compared to each other. This approach, however, also resulted in the unequal representation of the 183 structures in the network. Therefore we treated the overall quaternary organization of proteins with multiple chains as a single structure. Biological, ligand, and domain information were obtained using the RESTful web service interface provided by the RCSB. Only the biologically-significant transition metals were considered when painting the networks by bound metal. The Taxonomy Database from the NCBI was used to classify species into their respective domains and phyla. For the sequence similarity network, UniProt was used to map the PDB IDs used in the structure similarity network to their respective sequences in the UniProtKB database.

### Building the PSNs

Pythoscape was integral in the construction of the network. Pythoscape (1) imported sequences and structures into a database, (2) deployed TM-align and BLASTp for edge calculations, and (3) exported the completed database as a Cytoscape network file. Because TM-Align edge scores are directional (i.e., the score for the edge from A to B might be different than the score from B to A), edges were filtered based on the average of the two scores. Cytoscape 3.0 was used for network visualization in the as well as edge filtering. The Organic Layout was used as described in Atkinson, Babbitt et al. To construct the structure network composed only of domains, a script was written for PyMOL to extract and save the parts of the PDB files that had a domain as defined as by the RCSB. Domains outside of the PFAM cupin clan were manually removed from the network. Networks were visualized using two thresholds in order to illustrate effects edge stringency had on certain clusters. TM-scores of 0.53 and 0.65 were used as thresholds for the structure networks, and E-values of 1×10^−3.5^ and 1×10^−6.0^ were used as thresholds for the sequence networks. These values were selected based on overall visual appeal of the resulting layout in Cytoscape.

## Supporting Information

File S1 **Contains:** Table S1. Iron-containing structures used in the networks. Table S2. Nickel-containing structures used in the networks.Table S3. Zinc-containing structures used in the networks. Table S4. Manganese-containing structures used in the networks. Table S5. Structures used in the networks which contain copper, mercury, and cadmium, respectively. Table S6. Structures used in the networks that contain multiple metals. Table S7. Structures used in the networks that contain no transition metal.
(DOCX)
