# Supplementary material for: Protein Similarity Networks Reveal Relationships among Sequence, Structure, and Function within the Cupin Superfamily
Source: PLoS One. 2013 Sep 6;8(9):e74477. doi: 10.1371/journal.pone.0074477 (PMC3765361; doi:10.1371/journal.pone.0074477)
Supplement: File S1 — Contains: Table S1. Iron-containing structures used in the networks. Table S2. Nickel-containing structures used in the networks.Table S3. Zinc-containing structures used in the networks. Table S4. Manganese-containing structures used in the networks. Table S5. Structures used in the networks which contain copper, mercury, and cadmium, respectively. Table S6. Structures used in the networks that contain multiple metals. Table S7. Structures used in the networks that contain no transition metal. (DOCX) [file pone.0074477.s001.docx]

**File S1** Combined Supporting Information Files

**Table S1.** Iron-containing structures used in the networks.

| Protein | Species | PBD code | Ref. |
| --- | --- | --- | --- |
| 1-aminocyclopropane-1-carboxylate oxidase | Petunia hybrid | 1wa6 | [[1](#_ENREF_1)] |
| Deacetoxycephalosporin C synthase | *Streptomyces clavuligerus* | 1w28 | [[2](#_ENREF_2)**]** |
| Isopenicillin N synthase | *Emericella nidulans* | 1odm | [[3](#_ENREF_3)] |
| Hypoxia-inducible factor prolyl hydroxylase (PHD2) | *Homo sapiens* | 2g19 | [[4](#_ENREF_4)] |
| Ectoine hydroxylase (EctD) | *Virgibacillus salexigens* | 3emr | [[5](#_ENREF_5)] |
| putative cytoplasmic protein | *Salmonella typhimurium* | 2csg |  |
| syringomycin biosynthesis enzyme 2 | *Pseudomonas syringae* | 2fct | [[6](#_ENREF_6)] |
| uncharacterized protein Q2CBJ1_9RHOB | *Oceanicola granulosus* | 2rg4 |  |
| protein product of AT3G21360, *Arabidopsis thaliana* | *Arabidopsis thaliana* | 1y0z | [[7](#_ENREF_7)] |
| 3-hydroxyanthranilate-3,4-dioxygenase | *Cupriavidus metallidurans* | 1yfu | [[8](#_ENREF_8)] |
| 3-hydroxyanthranilate-3,4-dioxygenase | *Bos taurus* | 3fe5 | [[9](#_ENREF_9)] |
| Putative cysteine dioxygenase | *Bacillus subtilis* | 3eqe |  |
| Gab protein | *Escherichia coli* | 1jr7 | [[10](#_ENREF_10),[11](#_ENREF_11)] |
| Hydroxypropylphosphonic acid epoxidase (FeHPPE) | *Streptomyces wedmorensis* | 1zz7 | [[12](#_ENREF_12)] |
| Gentisate 1,2-dioxygenase | *Pseudaminobacter salicylatoxidans* | 3njz | [[13](#_ENREF_13)] |
| Gentisate 1,2-dioxygenase | *Silicibacter pomeroyi* | 3bu7 | [[14](#_ENREF_14)] |
| Quercetin 2,3-dioxygenase | *Bacillus subtilis* | 1y3t | [[15](#_ENREF_15)] |
| CytC3 | *Streptomyces sp*. | 3gja | [[16](#_ENREF_16)] |
| Putative asparaginyl hydroxylase | *Bacillus subtilis* | 1vrb |  |
| factor inhibiting HIF1 | *Homo sapiens* | 2y0i | [[17](#_ENREF_17)] |
| Protein fto | *Homo sapiens* | 3lfm | [[18](#_ENREF_18)] |
| JmjC domain-containing histone demethylation protein 1A | *Homo sapiens* | 2yu2 |  |
| JmjC domain-containing histone demethylation protein 3D | *Homo sapiens* | 3dxu |  |
| Alkylated repair protein ALKB homolog 3 | *Homo sapiens* | 2iuw | [[19](#_ENREF_19)] |
| PHD finger protein 2 | *Homo sapiens* | 3pu8 | [[20](#_ENREF_20)] |
| catalytic domain of PHD finger protein 8 | *Homo sapiens* | 3k3n | [[21](#_ENREF_21)] |
| PKHD-type hydroxylase TPA1 | *Saccharomyces cerevisiae* | 3kt4 | [[22](#_ENREF_22)] |
| Phytanoyl-CoA dioxygenase domain-containing protein 1 | *Homo sapiens* | 3obz |  |
| CurA halogenase | *Lyngbya majuscula* | 3nnf | [[23](#_ENREF_23)] |
| 1-deoxypentalenic acid 11-beta hydroxylase | *Streptomyces avermitilis* | 2rdn | [[24](#_ENREF_24)] |
| Alpha-ketoglutarate-dependent taurine dioxygenase | *Mycobacterium avium* | 3r1j |  |
| Taurine catabolism dioxygenase TauD | *Mycobacterium marinum* | 3swt |  |
| L-arginine beta- hydroxylase | *Streptomyces vinaceus* | 2wbp | [[25](#_ENREF_25)] |
| Putative alkylsulfatase AtsK | *Pseudomonas putida* | 1oii | [[26](#_ENREF_26)] |
| Carbapenem synthase (CarC) | *Pectobacterium carotovorum* | 1nx8 | [[27](#_ENREF_27)] |
| Taurine/alpha-ketoglutarate dioxygenase (TauD) | *Escherichia coli* | 1os7 | [[28](#_ENREF_28)] |
| Phosphoglucose isomerase | *Thermococcus litoralis* | 1j3p | [[29](#_ENREF_29)] |
| Pirin | *Homo sapiens*, | 1j1l | [[30](#_ENREF_30)] |
| Pirin-like protein | *Geobacillus kaustophilus* | 2p17 |  |
| Homogentisate dioxygenase | *Homo sapiens* | 1ey2 | [[31](#_ENREF_31)] |
| Anthocyanidin synthase | *Arabidopsis thaliana* | 1gp6 | [[32](#_ENREF_32)] |
| Cysteine dioxygenase type I | *Ralstonia eutropha* | 2gm6 |  |
| Cysteine dioxygenase type 1 | *Homo sapiens* | 2ic1 | [[33](#_ENREF_33)] |
| Bacterial cysteine dioxygenase | *Pseudomonas aeruginosa* | 3uss |  |
| Putative gentisate 1,2-dioxygenase | *Escherichia coli* | 2d40 | [[34](#_ENREF_34)] |
| Clavaminate synthase | *Stretomyces clavuligerus* | 1dry | [[35](#_ENREF_35)] |
| Type II Proline 3-hydroxylase | *Stretomyces sp*., | 1e5s | [[36](#_ENREF_36)] |
| Phytanoyl-CoA 2-hydroxylase | *Homo sapiens* | 2a1x | [[37](#_ENREF_37)] |

**Table S2.** Nickel-containing structures used in the networks.

| Protein | Species | PBD code | Ref. |
| --- | --- | --- | --- |
| 3-hydroxyanthranilate-3,4-dioxygenase | *Saccharomyces cerevisiae* | 1zvf |  |
| 3-hydroxyanthranilate-3,4-dioxygenase | *Homo sapiens* | 2qnk |  |
| Acireductone dioxygenase | *Klebsiella pneumoniae* | 1zrr | [[38](#_ENREF_38)] |
| Acireductone dioxygenase | *Mus musculus* | 1vr3 | [[39](#_ENREF_39)] |
| Cysteine dioxygenase type 1 | *Mus musculus* | 2q4s | [[40](#_ENREF_40),[41](#_ENREF_41)] |
| hypothetical protein (YP_555756.1) | *Burkholderia xenovorans* | 2o8q |  |
| Cupin 2 conserved barrel domain protein | *Sphaerobacter thermophilus* | 4e2g |  |
| Glucose-6-phosphate isomerase | *Pyrococcus furiosus* | 1qxj | [[42](#_ENREF_42)] |
| 2-oxoglutarate oxygenase | *Homo sapiens* | 2w2i |  |
| JmjC domain-containing protein C2orf60 | *Homo sapiens* | 3al6 | [[43](#_ENREF_43)] |
| Lysine-specific demethylase 7 | *Homo sapiens* | 3u78 | [[44](#_ENREF_44)] |
| Lysine-specific demethylase 8 | *Homo sapiens* | 4aap |  |
| Lysine-specific demethylase NO66 | *Homo sapiens* | 4diq |  |
| Uncharacterized IolB-like protein | *Salmonella typhimurium LT2* | 2qjv |  |
| Cupin 2, conserved barrel domain protein | *Shewanella frigidmarina* | 3d82 |  |
| DNA damage-responsive transcriptional repressor RPH1 | *Saccharomyces cerevisiae* | 3opt | [[45](#_ENREF_45)] |
| PKHD-type hydroxylase Sbal_3634 | *Shewanella baltica* | 3dkq |  |

**Table S3.** Zinc-containing structures used in the networks.

| Protein | Species | PBD code | Ref. |
| --- | --- | --- | --- |
| Aspartate beta-hydroxylase | *Homo sapiens* | 3rcq |  |
| hypothetical protein BP2299 | *Bordetella pertussis* | 2fqp |  |
| Auxin binding protein | *Zea mays* | 1lrh | [[46](#_ENREF_46)] |
| Predicted protein, algal prolyl 4-hydroxylase | *Chlamydomonas reinhardtii* | 3gze | [[47](#_ENREF_47)] |
| Uncharacterized protein year | *Escherichia coli* | 3bb6 |  |
| transcriptional regulator, HTH_3 family | *Vibrio cholerae* | 1y9q |  |
| Cupin 2, conserved barrel domain protein | *Halorhodospira halophila* | 3ibm |  |
| Anti-ECFsigma factor, ChrR | *Marinobacter aquaeolei* | 3o14 |  |
| RpoE, ECF SigE | *Rhodobacter sphaeroides* | 2z2s | [[48](#_ENREF_48)] |
| 4-deoxy-L-threo-5-hexosulose-uronate ketol-isomerase | *Escherichia coli* | 1xru | [[49](#_ENREF_49)] |
| Mannose-6-phosphate isomerase | *Bacillus subtilis* | 1qwr |  |
| Mannose-6-phosphate isomerase | *Salmonella typhimurium* | 3h1m | [[50](#_ENREF_50)] |
| Tetracenomycin polyketide synthesis protein | *Xanthomonas campestris* | 3h50 | [[51](#_ENREF_51)] |
| Polyketide cyclase RemF | *Streptomyces resistomycificus* | 3ht2 | [[52](#_ENREF_52)] |
| Phosphomannose isomerase | *Candida albicans* | 1pmi | [[53](#_ENREF_53)] |
| Uncharacterized cupin protein | *Bacteroides fragilis* | 3cew |  |
| Gamma-butyrobetaine dioxygenase | *Homo sapiens* | 3n6w | [[54](#_ENREF_54)] |

**Table S4.** Manganese-containing structures used in the networks.

| Protein | Species | PBD code | Ref. |
| --- | --- | --- | --- |
| Oxalate decarboxylase | *Bacillus subtilis* | 1uw8 | [[55](#_ENREF_55)] |
| SLL1358 protein | *Synechocystis sp*. | 2vqa | [[56](#_ENREF_56)] |
| Cupin 2 conserved barrel domain protein | *Rhodopseudomonas palustris* | 3kgz |  |
| uncharacterized protein Rru_A2000 | *Rhodospirillum rubrum* | 3jzv |  |
| Ureidoglycine aminohydrolase | Arabidopsis thaliana | 4e2q | [[57](#_ENREF_57)] |
| Hypothetical protein TM1287 | *Thermatoga maritima* | 1o4t | [[58](#_ENREF_58)] |
| Hypothetical protein TM1459 | *Thermatoga maritima* | 1vj2 | [[59](#_ENREF_59)] |
| D-lyxose isomerase | *Escherichia coli* O157:H7 | 3kmh | [[60](#_ENREF_60)] |
| Alpha-ketoglutarate-dependent dioxygenase alkB homolog 2 | *Homo sapiens* | 3buc | [[61](#_ENREF_61)] |
| Germin (Oxalate oxidase) | *Hordeum vulgare* | 2et1 | [[62](#_ENREF_62)] |

**Table S5.** Structures used in the networks which contain copper, mercury, and cadmium, respectively.

| Protein | Species | Metal ion | PBD code | Ref. |
| --- | --- | --- | --- | --- |
| Quercetin 2,3-dioxygenase | *Aspergillus japonicus* | Cu | 1juh | [[63](#_ENREF_63)] |
| Hypothetical protein HI0227 | *Haemophilus influenzae* | Hg | 1jop | [[64](#_ENREF_64)] |
| Protein yhhW | *Escherichia coli* | Cd | 1tq5 | [[65](#_ENREF_65)] |

**Table S6.** Structures used in the networks that contain multiple metals.

| Protein | Species | PBD code | Ref. |
| --- | --- | --- | --- |
| Bifunctional arginine demethylase and lysyl-hydroxylase JMJD6 | *Homo sapiens* | 3ld8 | [[66](#_ENREF_66)] |
| Putative uncharacterized protein | *Caenorhabditis elegans* | 3n9l | [[67](#_ENREF_67)] |
| Lysine-specific demethylase 6B | *Homo sapiens* | 2xue | [[68](#_ENREF_68)] |
| Jumonji domain-containing protein 2A | *Homo sapiens* | 2gp3 | [[69](#_ENREF_69)] |
| Bacilysin biosynthesis protein bacB | *Bacillus subtilis* | 3h7j | [[70](#_ENREF_70),[71](#_ENREF_71)] |
| MYC-induced nuclear antigen | *Homo sapiens* | 2xdv |  |
| Alkylated DNA repair protein alkB homolog 8 | *Homo sapiens* | 3thp | [[72](#_ENREF_72)] |
| Uncharacterized protein Ism_01780 | *Roseovarius nubinhibens* | 3bvc |  |
| Lysine-specific demethylase 6A | *Homo sapiens* | 3avr | [[73](#_ENREF_73)] |
| JmjC domain-containing histone demethylation protein 3A | *Homo sapiens* | 2oq6 | [[74](#_ENREF_74)] |
| JMJD2C catalytic domain | *Homo sapiens* | 2xml |  |
| uncharacterized protein RPA4178 | *Rhodopseudomonas palustris* | 3lag |  |

**Table S7.** Structures used in the networks that contain no transition metal.

| Protein | Species | PBD code | Ref. |
| --- | --- | --- | --- |
| Regulatory protein AraC | *Escherichia coli* | 2ara | [[66](#_ENREF_66),[75](#_ENREF_75)] |
| Ara h 3, a major allergen in peanut | *Arachis hypogaea* | 3c3v | [[76](#_ENREF_76)] |
| Allergen Ara h 1, clone P41B | *Arachis hypogaea* | 3s7 | [[77](#_ENREF_77)] |
| Beta-conglycinin | *Glycine max* | 1uij | [[78](#_ENREF_78)] |
| Proglycinin | *Glycine max* | 1fxz | [[79](#_ENREF_79)] |
| Glycinin | *Glycine max* | 1od5 | [[80](#_ENREF_80)] |
| Prolegumin | *Pisum sativum* | 3ksc | [[81](#_ENREF_81)] |
| 11S globulin subunit beta | *Cucurbita maxima* | 2e9q | [[81](#_ENREF_81)] |
| 11S globulin seed storage protein | *Amaranthus hypochondriacus* | 3qac |  |
| hypothetical protein TM1010 | *Thermatoga maritima* | 2f4p |  |
| uncharacterized protein | *Methylibium petroleiphilum* (strain PM1), | 3pl0 |  |
| Alpha-ketoglutarate-dependent taurine dioxygenase | *Pseudomonas putida* | 3v15 | [[82](#_ENREF_82)] |
| Putative uncharacterized protein | *Leishmania major* | 3m3i |  |
| Uncharacterized protein | *Bordetella bronchiseptica* | 3bcw |  |
| uncharacterized RmlC-like cupin | *Ralstonia eutropha* | 3ebr |  |
| uncharacterized protein with RmlC-like cupin fold | *Ralstonia eutropha* | 3fjs |  |
| Cupin 2, conserved barrel domain protein | *Novosphingobium aromaticivorans* | 3es1 |  |
| uncharacterized protein DUF861 with a RmlC-like cupin fold | *Agrobacterium fabrum* | 3es4 |  |
| Putative uncharacterized protein ( HutD) | *Pseudomas fluorescens* | 3esg |  |
| Prunin | *prunus dulcis* | 3ehk | [[83](#_ENREF_83)] |
| Pyoverdine biosynthesis protein PvcB | *Pseudomonas aeruginosa* | 3eat | [[84](#_ENREF_84)] |
| UPF0345 protein ACIAD0356 | *Acinetobacter sp*. | 3hqx |  |
| Tellurite resistance protein B | *Vibrio fisheri* | 3dl3 |  |
| Tellurite resistance protein tehB homolog | *Haemophilus influenza* | 3m70 |  |
| sugar phosphate isomerase | *Ruegeria pomeroyi* | 3i7d |  |
| Prolyl 4-hydroxylase, alpha subunit domain protein | *Bacillus anthracis* | 3itq | [[85](#_ENREF_85)] |
| 7S globulin-1 | *Vigna angularis* | 2ea7 | [[86](#_ENREF_86)] |
| Putative oxygenase | *Shewanella oneidensis* | 3on7 |  |
| Hypothetical protein ybiU | *Escherichia coli* | 2dbi |  |
| Putative oxygenase | *Caulobacter crescentus* | 3oox |  |
| Phaseolin | *Phaseolus vulgaris* | 2phl | [[87](#_ENREF_87)] |
| Hypothetical protein | *Neisseria meningitidis* | 2i45 |  |
| Procruciferin | *Brassica napus* | 3kgl | [[81](#_ENREF_81)] |
| alpha prime subunit of beta-conglycinin | *Glycine max* | 1uik | [[88](#_ENREF_88)] |
| Hypothetical protein MJ0764 | *Methanocaldococcus jannaschii* | 2b8m |  |
| Putative sugar phosphate isomerase | *Acidithiobacillus ferrooxidans* | 3l2h |  |
| Seed storage protein | *Vigna radiata* | 2cv6 | [[89](#_ENREF_89)] |
| Cupin 2, conserved barrel | *Leptotrichia buccalis* | 3rns |  |
| Cupin 2, conserved barrel domain protein | *Shewanella frigidimarina* | 2pfw |  |
| Rmlc-like cupin protein (dde_2303) | *Desulfovibrio desulfuricans* | 2q30 |  |
| Hypothetical protein | *Ralstonia eutropha* | 2opk |  |
| probable antibiotics synthesis protein | *Thermus thermophilus* | 1v70 |  |
| 4-deoxy-L-threo-5-hexosulose-uronate ketol-isomerase 1 | *Enterococcus faecalis* | 1ywk |  |
| conserved hypothetical protein | *Enterococcus faecalis* | 1sef |  |
| hypothetical protein SPy1581 | *Streptococcus pyogenes* | 1yhf |  |
| uncharacterized conserved protein | *Klebsiella pneumonniae* | 3h8u |  |
| Putative glyoxylate-induced protein | *Pseudomonas aeruginosa* | 1sq4 |  |
| BH2720 protein | *Bacillus halodurans* | 2oa2 |  |
| conserved hypothetical protein | *Deinicoccus radiodurans* | 1sfn |  |
| Hypothetical protein ylbA | *Escherichia coli* | 1rc6 |  |
| PSPTO_0244 | *Pseudomonas syringae* | 3myx |  |
| Hypothetical protein TM1112 | *Thermatoga maritima* | 1o5u | [[90](#_ENREF_90)] |
| hypothetical protein Atu3615 | *Agrobacterium tumefaciens* | 1znp |  |
| Putative uncharacterized protein | *Branchiostoma belcheri tsingtauense* | 3loi | [[91](#_ENREF_91)] |
| hypothetical protein SO0799 | *Shewanella oneidensis* | 1yud |  |
| dTDP-4-keto-6-deoxy-glucose-5-epimerase EvaD | *Amycolatopsis orientalis* | 1ofn | [[92](#_ENREF_92)] |
| dTDP-4-dehydrorhamnose 3,5-epimerase | *Bacillus anthracis* | 3ryk |  |
| dTDP-4-dehydrorhamnose 3,5-epimerase | *Streptococcus suis* | 1nxm | [[93](#_ENREF_93)] |
| dTDP-4-dehydrorhamnose 3,5-epimerase | *Sulfolobus tokodaii* | 1wlt |  |
| dTDP-4-dehydrorhamnose 3,5-epimerase | *Mycobacterium tuberculosis* | 1upi | [[94](#_ENREF_94)] |
| dTDP-6-deoxy-D-xylo-4-hexulose 3,5-epimerase | *Pseudomonas aeruginosa* | 1rtv | [[95](#_ENREF_95)] |
| dTDP-4-dehydrorhamnose 3,5-epimerase | *Salmonella typhimurium* | 1dzr | [[96](#_ENREF_96)] |
| (dTDP)-4-keto-6-deoxy-d-hexulose 3, 5-epimerase | *Methanobaterium thermoautotrophicum* | 1ep0 | [[97](#_ENREF_97)] |
| UPF0345 protein VPA0057 | *Vibrio parahaemolyticus* | 2oyz |  |
| Ethanolamine utilization protein eutQ | *Salmonella typhimurium* | 2pyt |  |
| Uncharacterized protein | *Rhizobium leguminosarum* | 3lwc |  |
| Uncharacterized protein | *Nostoc punctiforme* | 2qdr |  |
| dTDP-6-deoxy-3,4-keto-hexulose isomerase | *Aneurinibacillus thermoaerophilus* | 2pa7 | [[98](#_ENREF_98)] |
| conserved hypothetical protein | *Pseudomonas aeruginosa* | 1yll |  |
| protein Mif2p | *Saccharomyces cerevisiae* | 2vpv | [[99](#_ENREF_99)] |
| protein YhaK | Escherichia coli | 2vec | [[100](#_ENREF_100)] |
| mannosephosphate isomerase, putative | Archaeoglobus fulgidus | 1zx5 |  |
| Canavalin/vinculin | Canavalia ensiformis | 1dgw | [[101](#_ENREF_101)] |
| YML079w | Saccharomyces cerevisiae | 1xe7 | [[102](#_ENREF_102)] |
| 4-Keto-6-deoxy sugar Epimerase (NovW) | Streptomyces spheroides | 2c0z | [[103](#_ENREF_103)] |
| Cupin 2 conserved barrel | Desulfitobacterium hafniense | 2ozj |  |

**References**

1. Zhang Z, Ren JS, Clifton IJ, Schofield CJ (2004) Crystal structure and mechanistic implications of 1-aminocyclopropane-1-carboxylic acid oxidase--the ethylene-forming enzyme. Chem Biol 11: 1383-1394.

2. Oster LM, van Scheltinga AC, Valegard K, Hose AM, Dubus A, et al. (2004) Conformational flexibility of the C terminus with implications for substrate binding and catalysis revealed in a new crystal form of deacetoxycephalosporin C synthase. J Mol Biol 343: 157-171.

3. Elkins JM, Rutledge PJ, Burzlaff NI, Clifton IJ, Adlington RM, et al. (2003) Crystallographic studies on the reaction of isopenicillin N synthase with an unsaturated substrate analogue. Org Biomol Chem 1: 1455-1460.

4. McDonough MA, Li V, Flashman E, Chowdhury R, Mohr C, et al. (2006) Cellular oxygen sensing: Crystal structure of hypoxia-inducible factor prolyl hydroxylase (PHD2). Proc Natl Acad Sci U S A 103: 9814-9819.

5. Reuter K, Pittelkow M, Bursy J, Heine A, Craan T, et al. (2010) Synthesis of 5-hydroxyectoine from ectoine: crystal structure of the non-heme iron(II) and 2-oxoglutarate-dependent dioxygenase EctD. PLoS One 5: e10647.

6. Blasiak LC, Vaillancourt FH, Walsh CT, Drennan CL (2006) Crystal structure of the non-haem iron halogenase SyrB2 in syringomycin biosynthesis. Nature 440: 368-371.

7. Bitto E, Bingman CA, Allard ST, Wesenberg GE, Aceti DJ, et al. (2005) The structure at 2.4 A resolution of the protein from gene locus At3g21360, a putative Fe(II)/2-oxoglutarate-dependent enzyme from Arabidopsis thaliana. Acta Crystallogr Sect F Struct Biol Cryst Commun 61: 469-472.

8. Zhang Y, Colabroy KL, Begley TP, Ealick SE (2005) Structural studies on 3-hydroxyanthranilate-3,4-dioxygenase: the catalytic mechanism of a complex oxidation involved in NAD biosynthesis. Biochemistry 44: 7632-7643.

9. Dilovic I, Gliubich F, Malpeli G, Zanotti G, Matkovic-Calogovic D (2009) Crystal structure of bovine 3-hydroxyanthranilate 3,4-dioxygenase. Biopolymers 91: 1189-1195.

10. Chance MR, Bresnick AR, Burley SK, Jiang JS, Lima CD, et al. (2002) Structural genomics: a pipeline for providing structures for the biologist. Protein Sci 11: 723-738.

11. Lohkamp B, Dobritzsch D (2008) A mixture of fortunes: the curious determination of the structure of Escherichia coli BL21 Gab protein. Acta Crystallogr D Biol Crystallogr 64: 407-415.

12. Higgins LJ, Yan F, Liu P, Liu HW, Drennan CL (2005) Structural insight into antibiotic fosfomycin biosynthesis by a mononuclear iron enzyme. Nature 437: 838-844.

13. Ferraroni M, Matera I, Steimer L, Burger S, Scozzafava A, et al. (2012) Crystal structures of salicylate 1,2-dioxygenase-substrates adducts: A step towards the comprehension of the structural basis for substrate selection in class III ring cleaving dioxygenases. J Struct Biol 177: 431-438.

14. Chen J, Li W, Wang M, Zhu G, Liu D, et al. (2008) Crystal structure and mutagenic analysis of GDOsp, a gentisate 1,2-dioxygenase from Silicibacter pomeroyi. Protein Sci 17: 1362-1373.

15. Gopal B, Madan LL, Betz SF, Kossiakoff AA (2005) The crystal structure of a quercetin 2,3-dioxygenase from Bacillus subtilis suggests modulation of enzyme activity by a change in the metal ion at the active site(s). Biochem 44: 193-201.

16. Wong C, Fujimori DG, Walsh CT, Drennan CL (2009) Structural analysis of an open active site conformation of nonheme iron halogenase CytC3. J Am Chem Soc 131: 4872-4879.

17. Yang M, Chowdhury R, Ge W, Hamed RB, McDonough MA, et al. (2011) Factor-inhibiting hypoxia-inducible factor (FIH) catalyses the post-translational hydroxylation of histidinyl residues within ankyrin repeat domains. FEBS J 278: 1086-1097.

18. Han Z, Niu T, Chang J, Lei X, Zhao M, et al. (2010) Crystal structure of the FTO protein reveals basis for its substrate specificity. Nature 464: 1205-1209.

19. Sundheim O, Vagbo CB, Bjoras M, Sousa MM, Talstad V, et al. (2006) Human ABH3 structure and key residues for oxidative demethylation to reverse DNA/RNA damage. EMBO J 25: 3389-3397.

20. Horton JR, Upadhyay AK, Hashimoto H, Zhang X, Cheng X (2011) Structural basis for human PHF2 Jumonji domain interaction with metal ions. J Mol Biol 406: 1-8.

21. Yu L, Wang Y, Huang S, Wang J, Deng Z, et al. (2010) Structural insights into a novel histone demethylase PHF8. Cell Res 20: 166-173.

22. Kim HS, Kim HL, Kim KH, Kim do J, Lee SJ, et al. (2010) Crystal structure of Tpa1 from Saccharomyces cerevisiae, a component of the messenger ribonucleoprotein complex. Nucleic Acids Res 38: 2099-2110.

23. Khare D, Wang B, Gu L, Razelun J, Sherman DH, et al. (2010) Conformational switch triggered by alpha-ketoglutarate in a halogenase of curacin A biosynthesis. Proc Natl Acad Sci U S A 107: 14099-14104.

24. You Z, Omura S, Ikeda H, Cane DE, Jogl G (2007) Crystal structure of the non-heme iron dioxygenase PtlH in pentalenolactone biosynthesis. J Biol Chem 282: 36552-36560.

25. Helmetag V, Samel SA, Thomas MG, Marahiel MA, Essen LO (2009) Structural basis for the erythro-stereospecificity of the L-arginine oxygenase VioC in viomycin biosynthesis. FEBS J 276: 3669-3682.

26. Muller I, Kahnert A, Pape T, Sheldrick GM, Meyer-Klaucke W, et al. (2004) Crystal structure of the alkylsulfatase AtsK: insights into the catalytic mechanism of the Fe(II) alpha-ketoglutarate-dependent dioxygenase superfamily. Biochemistry 43: 3075-3088.

27. Clifton IJ, Doan LX, Sleeman MC, Topf M, Suzuki H, et al. (2003) Crystal structure of carbapenem synthase (CarC). J Biol Chem 278: 20843-20850.

28. O'Brien JR, Schuller DJ, Yang VS, Dillard BD, Lanzilotta WN (2003) Substrate-induced conformational changes in Escherichia coli taurine/alpha-ketoglutarate dioxygenase and insight into the oligomeric structure. Biochemistry 42: 5547-5554.

29. Jeong JJ, Fushinobu S, Ito S, Jeon BS, Shoun H, et al. (2003) Characterization of the cupin-type phosphoglucose isomerase from the hyperthermophilic archaeon Thermococcus litoralis. FEBS Lett 535: 200-204.

30. Pang H, Bartlam M, Zeng Q, Miyatake H, Hisano T, et al. (2004) Crystal structure of human pirin: an iron-binding nuclear protein and transcription cofactor. J Biol Chem 279: 1491-1498.

31. Titus GP, Mueller HA, Burgner J, Rodriguez De Cordoba S, Penalva MA, et al. (2000) Crystal structure of human homogentisate dioxygenase. Nat Struct Biol 7: 542-546.

32. Wilmouth RC, Turnbull JJ, Welford RW, Clifton IJ, Prescott AG, et al. (2002) Structure and mechanism of anthocyanidin synthase from Arabidopsis thaliana. Structure 10: 93-103.

33. Ye S, Wu X, Wei L, Tang D, Sun P, et al. (2007) An insight into the mechanism of human cysteine dioxygenase. Key roles of the thioether-bonded tyrosine-cysteine cofactor. J Biol Chem 282: 3391-3402.

34. Adams MA, Singh VK, Keller BO, Jia Z (2006) Structural and biochemical characterization of gentisate 1,2-dioxygenase from Escherichia coli O157:H7. Mol Microbiol 61: 1469-1484.

35. Zhang Z, Ren J, Stammers DK, Baldwin JE, Harlos K, et al. (2000) Structural origins of the selectivity of the trifunctional oxygenase clavaminic acid synthase. Nat Struct Biol 7: 127-133.

36. Clifton IJ, Hsueh LC, Baldwin JE, Harlos K, Schofield CJ (2001) Structure of proline 3-hydroxylase. Evolution of the family of 2-oxoglutarate dependent oxygenases. Eur J Biochem 268: 6625-6636.

37. McDonough MA, Kavanagh KL, Butler D, Searls T, Oppermann U, et al. (2005) Structure of human phytanoyl-CoA 2-hydroxylase identifies molecular mechanisms of Refsum disease. J Biol Chem 280: 41101-41110.

38. Pochapsky TC, Pochapsky SS, Ju T, Hoefler C, Liang J (2006) A refined model for the structure of acireductone dioxygenase from Klebsiella ATCC 8724 incorporating residual dipolar couplings. Journal of Biomolecular NMR 34: 117-127.

39. Xu Q, Schwarzenbacher R, Krishna SS, McMullan D, Agarwalla S, et al. (2006) Crystal structure of acireductone dioxygenase (ARD) from Mus musculus at 2.06 angstrom resolution. Proteins 64: 808-813.

40. Levin EJ, Kondrashov DA, Wesenberg GE, Phillips GN, Jr. (2007) Ensemble refinement of protein crystal structures: validation and application. Structure 15: 1040-1052.

41. McCoy JG, Bailey LJ, Bitto E, Bingman CA, Aceti DJ, et al. (2006) Structure and mechanism of mouse cysteine dioxygenase. Proc Natl Acad Sci U S A 103: 3084-3089.

42. Swan MK, Solomons JT, Beeson CC, Hansen T, Schonheit P, et al. (2003) Structural evidence for a hydride transfer mechanism of catalysis in phosphoglucose isomerase from Pyrococcus furiosus. J Biol Chem 278: 47261-47268.

43. Kato M, Araiso Y, Noma A, Nagao A, Suzuki T, et al. (2011) Crystal structure of a novel JmjC-domain-containing protein, TYW5, involved in tRNA modification. Nucleic Acids Res 39: 1576-1585.

44. Upadhyay AK, Rotili D, Han JW, Hu R, Chang Y, et al. (2012) An analog of BIX-01294 selectively inhibits a family of histone H3 lysine 9 Jumonji demethylases. J Mol Biol 416: 319-327.

45. Chang Y, Wu J, Tong XJ, Zhou JQ, Ding J (2011) Crystal structure of the catalytic core of Saccharomyces cerevesiae histone demethylase Rph1: insights into the substrate specificity and catalytic mechanism. Biochem J 433: 295-302.

46. Woo EJ, Marshall J, Bauly J, Chen JG, Venis M, et al. (2002) Crystal structure of auxin-binding protein 1 in complex with auxin. Embo J 21: 2877-2885.

47. Koski MK, Hieta R, Hirsila M, Ronka A, Myllyharju J, et al. (2009) The crystal structure of an algal prolyl 4-hydroxylase complexed with a proline-rich peptide reveals a novel buried tripeptide binding motif. J Biol Chem 284: 25290-25301.

48. Campbell EA, Greenwell R, Anthony JR, Wang S, Lim L, et al. (2007) A conserved structural module regulates transcriptional responses to diverse stress signals in bacteria. Mol Cell 27: 793-805.

49. Crowther RL, Georgiadis MM (2005) The crystal structure of 5-keto-4-deoxyuronate isomerase from Escherichia coli. Proteins 61: 680-684.

50. Sagurthi SR, Gowda G, Savithri HS, Murthy MR (2009) Structures of mannose-6-phosphate isomerase from Salmonella typhimurium bound to metal atoms and substrate: implications for catalytic mechanism. Acta Crystallogr D Biol Crystallogr 65: 724-732.

51. Axelrod HL, Kozbial P, McMullan D, Krishna SS, Miller MD, et al. (2010) Conformational changes associated with the binding of zinc acetate at the putative active site of XcTcmJ, a cupin from Xanthomonas campestris pv. campestris. Acta Crystallogr Sect F Struct Biol Cryst Commun 66: 1347-1353.

52. Silvennoinen L, Sandalova T, Schneider G (2009) The polyketide cyclase RemF from Streptomyces resistomycificus contains an unusual octahedral zinc binding site. FEBS Lett 583: 2917-2921.

53. Cleasby A, Wonacott A, Skarzynski T, Hubbard RE, Davies GJ, et al. (1996) The x-ray crystal structure of phosphomannose isomerase from Candida albicans at 1.7 angstrom resolution. Nat Struct Biol 3: 470-479.

54. Tars K, Rumnieks J, Zeltins A, Kazaks A, Kotelovica S, et al. (2010) Crystal structure of human gamma-butyrobetaine hydroxylase. Biochem Biophys Res Commun 398: 634-639.

55. Just VJ, Stevenson CE, Bowater L, Tanner A, Lawson DM, et al. (2004) A closed conformation of Bacillus subtilis oxalate decarboxylase OxdC provides evidence for the true identity of the active site. J Biol Chem 279: 19867-19874.

56. Tottey S, Waldron KJ, Firbank SJ, Reale B, Bessant C, et al. (2008) Protein-folding location can regulate manganese-binding versus copper- or zinc-binding. Nature 455: 1138-1142.

57. Shin I, Percudani R, Rhee S (2012) Structural and functional insights into (S)-ureidoglycine aminohydrolase, key enzyme of purine catabolism in Arabidopsis thaliana. J Biol Chem 287: 18796-18805.

58. Schwarzenbacher R, von Delft F, Jaroszewski L, Abdubek P, Ambing E, et al. (2004) Crystal structure of a putative oxalate decarboxylase (TM1287) from Thermotoga maritima at 1.95 A resolution. Proteins 56: 392-395.

59. Jaroszewski L, Schwarzenbacher R, von Delft F, McMullan D, Brinen LS, et al. (2004) Crystal structure of a novel manganese-containing cupin (TM1459) from Thermotoga maritima at 1.65 A resolution. Proteins 56: 611-614.

60. van Staalduinen LM, Park CS, Yeom SJ, Adams-Cioaba MA, Oh DK, et al. (2010) Structure-based annotation of a novel sugar isomerase from the pathogenic E. coli O157:H7. J Mol Biol 401: 866-881.

61. Yang CG, Yi C, Duguid EM, Sullivan CT, Jian X, et al. (2008) Crystal structures of DNA/RNA repair enzymes AlkB and ABH2 bound to dsDNA. Nature 452: 961-965.

62. Opaleye O, Rose RS, Whittaker MM, Woo EJ, Whittaker JW, et al. (2006) Structural and spectroscopic studies shed light on the mechanism of oxalate oxidase. J Biol Chem 281: 6428-6433.

63. Fusetti F, Schroter KH, Steiner RA, van Noort PI, Pijning T, et al. (2002) Crystal structure of the copper-containing quercetin 2,3-dioxygenase from Aspergillus japonicus. Structure 10: 259-268.

64. Teplyakov A, Obmolova G, Toedt J, Galperin MY, Gilliland GL (2005) Crystal structure of the bacterial YhcH protein indicates a role in sialic acid catabolism. J Bacteriol 187: 5520-5527.

65. Adams M, Jia Z (2005) Structural and biochemical analysis reveal pirins to possess quercetinase activity. J Biol Chem 280: 28675-28682.

66. Hong X, Zang J, White J, Wang C, Pan CH, et al. (2010) Interaction of JMJD6 with single-stranded RNA. Proc Natl Acad Sci U S A 107: 14568-14572.

67. Yang Y, Hu L, Wang P, Hou H, Lin Y, et al. (2010) Structural insights into a dual-specificity histone demethylase ceKDM7A from Caenorhabditis elegans. Cell Res 20: 886-898.

68. Kruidenier L, Chung CW, Cheng Z, Liddle J, Che K, et al. (2012) A selective jumonji H3K27 demethylase inhibitor modulates the proinflammatory macrophage response. Nature 488: 404-408.

69. Chen Z, Zang J, Whetstine J, Hong X, Davrazou F, et al. (2006) Structural insights into histone demethylation by JMJD2 family members. Cell 125: 691-702.

70. Rajavel M, Mitra A, Gopal B (2009) Role of Bacillus subtilis BacB in the synthesis of bacilysin. J Biol Chem 284: 31882-31892.

71. Rajavel M, Gopal B (2010) Analysis of multiple crystal forms of Bacillus subtilis BacB suggests a role for a metal ion as a nucleant for crystallization. Acta Crystallogr D Biol Crystallogr 66: 635-639.

72. Pastore C, Topalidou I, Forouhar F, Yan AC, Levy M, et al. (2012) Crystal structure and RNA binding properties of the RNA recognition motif (RRM) and AlkB domains in human AlkB homolog 8 (ABH8), an enzyme catalyzing tRNA hypermodification. J Biol Chem 287: 2130-2143.

73. Sengoku T, Yokoyama S (2011) Structural basis for histone H3 Lys 27 demethylation by UTX/KDM6A. Genes Dev 25: 2266-2277.

74. Ng SS, Kavanagh KL, McDonough MA, Butler D, Pilka ES, et al. (2007) Crystal structures of histone demethylase JMJD2A reveal basis for substrate specificity. Nature 448: 87-91.

75. Soisson SM, MacDougall-Shackleton B, Schleif R, Wolberger C (1997) Structural basis for ligand-regulated oligomerization of AraC. Science 276: 421-425.

76. Jin T, Guo F, Chen YW, Howard A, Zhang YZ (2009) Crystal structure of Ara h 3, a major allergen in peanut. Mol Immunol 46: 1796-1804.

77. Chruszcz M, Maleki SJ, Majorek KA, Demas M, Bublin M, et al. (2011) Structural and immunologic characterization of Ara h 1, a major peanut allergen. J Biol Chem 286: 39318-39327.

78. Maruyama N, Maruyama Y, Tsuruki T, Okuda E, Yoshikawa M, et al. (2003) Creation of soybean beta-conglycinin beta with strong phagocytosis-stimulating activity. Biochim Biophys Acta 1648: 99-104.

79. Adachi M, Takenaka Y, Gidamis AB, Mikami B, Utsumi S (2001) Crystal structure of soybean proglycinin A1aB1b homotrimer. J Mol Biol 305: 291-305.

80. Adachi M, Kanamori J, Masuda T, Yagasaki K, Kitamura K, et al. (2003) Crystal structure of soybean 11S globulin: glycinin A3B4 homohexamer. Proc Natl Acad Sci U S A 100: 7395-7400.

81. Tandang-Silvas MR, Fukuda T, Fukuda C, Prak K, Cabanos C, et al. (2010) Conservation and divergence on plant seed 11S globulins based on crystal structures. Biochim Biophys Acta 1804: 1432-1442.

82. Knauer SH, Hartl-Spiegelhauer O, Schwarzinger S, Hanzelmann P, Dobbek H (2012) The Fe(II)/alpha-ketoglutarate-dependent taurine dioxygenases from Pseudomonas putida and Escherichia coli are tetramers. FEBS J 279: 816-831.

83. Jin T, Albillos SM, Guo F, Howard A, Fu TJ, et al. (2009) Crystal structure of prunin-1, a major component of the almond (Prunus dulcis) allergen amandin. J Agric Food Chem 57: 8643-8651.

84. Drake EJ, Gulick AM (2008) Three-dimensional structures of Pseudomonas aeruginosa PvcA and PvcB, two proteins involved in the synthesis of 2-isocyano-6,7-dihydroxycoumarin. J Mol Biol 384: 193-205.

85. Culpepper MA, Scott EE, Limburg J (2010) Crystal structure of prolyl 4-hydroxylase from Bacillus anthracis. Biochemistry 49: 124-133.

86. Fukuda T, Maruyama N, Salleh MR, Mikami B, Utsumi S (2008) Characterization and crystallography of recombinant 7S globulins of Adzuki bean and structure-function relationships with 7S globulins of various crops. J Agric Food Chem 56: 4145-4153.

87. Lawrence MC, Izard T, Beuchat M, Blagrove RJ, Colman PM (1994) Structure of phaseolin at 2.2 A resolution. Implications for a common vicilin/legumin structure and the genetic engineering of seed storage proteins. J Mol Biol 238: 748-776.

88. Maruyama Y, Maruyama N, Mikami B, Utsumi S (2004) Structure of the core region of the soybean beta-conglycinin alpha' subunit. Acta Crystallogr D Biol Crystallogr 60: 289-297.

89. Itoh T, Garcia RN, Adachi M, Maruyama Y, Tecson-Mendoza EM, et al. (2006) Structure of 8Salpha globulin, the major seed storage protein of mung bean. Acta Crystallogr D Biol Crystallogr 62: 824-832.

90. McMullan D, Schwarzenbacher R, Jaroszewski L, von Delft F, Klock HE, et al. (2004) Crystal structure of a novel Thermotoga maritima enzyme (TM1112) from the cupin family at 1.83 A resolution. Proteins 56: 615-618.

91. Du Y, He YX, Gaowa S, Zhang X, Chen Y, et al. (2010) Crystal structures of the apo and GDP-bound forms of a cupin-like protein BbDUF985 from Branchiostoma belcheri tsingtauense. Proteins 78: 2714-2719.

92. Merkel AB, Temple GK, Burkart MD, Losey HC, Beis K, et al. (2002) Purification, crystallization and preliminary structural studies of dTDP-4-keto-6-deoxy-glucose-5-epimerase (EvaD) from Amycolatopsis orientalis, the fourth enzyme in the dTDP-L-epivancosamine biosynthetic pathway. Acta Crystallogr D Biol Crystallogr 58: 1226-1228.

93. Dong C, Major LL, Allen A, Blankenfeldt W, Maskell D, et al. (2003) High-resolution structures of RmlC from Streptococcus suis in complex with substrate analogs locate the active site of this class of enzyme. Structure 11: 715-723.

94. Kantardjieff KA, Kim CY, Naranjo C, Waldo GS, Lekin T, et al. (2004) Mycobacterium tuberculosis RmlC epimerase (Rv3465): a promising drug-target structure in the rhamnose pathway. Acta Crystallogr D Biol Crystallogr 60: 895-902.

95. Dong C, Major LL, Srikannathasan V, Errey JC, Giraud MF, et al. (2007) RmlC, a C3' and C5' carbohydrate epimerase, appears to operate via an intermediate with an unusual twist boat conformation. J Mol Biol 365: 146-159.

96. Giraud MF, Leonard GA, Field RA, Berlind C, Naismith JH (2000) RmlC, the third enzyme of dTDP-L-rhamnose pathway, is a new class of epimerase. Nat Struct Biol 7: 398-402.

97. Christendat D, Saridakis V, Dharamsi A, Bochkarev A, Pai EF, et al. (2000) Crystal structure of dTDP-4-keto-6-deoxy-D-hexulose 3,5-epimerase from Methanobacterium thermoautotrophicum complexed with dTDP. J Biol Chem 275: 24608-24612.

98. Davis ML, Thoden JB, Holden HM (2007) The x-ray structure of dTDP-4-keto-6-deoxy-D-glucose-3,4-ketoisomerase. J Biol Chem 282: 19227-19236.

99. Cohen RL, Espelin CW, De Wulf P, Sorger PK, Harrison SC, et al. (2008) Structural and functional dissection of Mif2p, a conserved DNA-binding kinetochore protein. Mol Biol Cell 19: 4480-4491.

100. Gurmu D, Lu J, Johnson KA, Nordlund P, Holmgren A, et al. (2009) The crystal structure of the protein YhaK from Escherichia coli reveals a new subclass of redox sensitive enterobacterial bicupins. Proteins 74: 18-31.

101. Ko TP, Kuznetsov YG, Malkin AJ, Day J, McPherson A (2001) X-ray diffraction and atomic force microscopy analysis of twinned crystals: rhombohedral canavalin. Acta Crystallogr D Biol Crystallogr 57: 829-839.

102. Zhou CZ, Meyer P, Quevillon-Cheruel S, De La Sierra-Gallay IL, Collinet B, et al. (2005) Crystal structure of the YML079w protein from Saccharomyces cerevisiae reveals a new sequence family of the jelly-roll fold. Protein Sci 14: 209-215.

103. Jakimowicz P, Tello M, Meyers CL, Walsh CT, Buttner MJ, et al. (2006) The 1.6-A resolution crystal structure of NovW: a 4-keto-6-deoxy sugar epimerase from the novobiocin biosynthetic gene cluster of Streptomyces spheroides. Proteins 63: 261-265.
